# Optimization of the *In Vitro* Proliferation of an Ancient Pear Tree Cultivar (‘Decana d’inverno’) through the Use of Neem Oil

**DOI:** 10.3390/plants12081593

**Published:** 2023-04-10

**Authors:** Luca Regni, Simona Lucia Facchin, Daniel Fernandes da Silva, Primo Proietti, Cristian Silvestri, Maurizio Micheli

**Affiliations:** 1 Department of Agricultural, Food and Environmental Sciences, University of Perugia, Borgo XX Giugno, 06121 Perugia, Italy; simonafacchin88@gmail.com (S.L.F.); primo.proietti@unipg.it (P.P.); maurizio.micheli@unipg.it (M.M.); 2 Campus Marechal Cândido Rondon, Universidade Estadual do Oeste do Paraná, Rua Pernambuco 1777, Cascavel 85819-110, PR, Brazil; daniel_eafi@yahoo.com.br; 3 Department of Agriculture and Forest Sciences (DAFNE), University of Tuscia, Via San Camillo de Lellis, 01100 Viterbo, Italy; silvestri.c@unitus.it

**Keywords:** micropropagation, fruit tree, culture media composition, natural substances

## Abstract

*In vitro* culture, ensuring rapid multiplication and production of plant material under aseptic conditions, represents an excellent tool for *ex-situ* conservation of tree species biodiversity and can be used for the conservation, among others, of endangered and rare crops. Among the *Pyrus communis* L. cultivars that have been abandoned over the years due to changed cultivation requirements, but which are still used today in breeding programs, there is the ‘Decana d’inverno’. Pear is generally considered a recalcitrant species for *in vitro* propagation due to weak multiplication rate, hyperhydricity, and susceptibility to phenolic oxidation. Therefore, the use of natural substances like neem oil (although little explored) represents one of the options to improve the *in vitro* plant’s tissue culture. In this context, the aim of the present work was to evaluate the effect of adding neem oil (0.1 and 0.5 m L L^−1^) to the growth substrate in order to optimise the *in vitro* culture of the ancient pear tree cultivar ‘Decana d’inverno’. The neem oil addition resulted in an increase in the number of shoots produced especially at both concentrations used. On the contrary, an increase in length of proliferated shoots was observed only with the addition of 0.1 mL L^−1^. The neem oil addition did not affect the explants viability, fresh and dry weights. Therefore, the present study demonstrated for the first time the possibility of using neem oil to optimise the *in vitro* culture of an ancient pear tree cultivar.

## 1. Introduction

The genus *Pyrus* is characterized by a high genetic variability with several species and thousands of cultivars that can be divided into two major groups, the Occidental (European) and the Oriental (Asian) pears [1]. Regarding the typology of pears produced, Asian pear cultivars are widely cultivated in China while European pear cultivars are produced in other countries [2].

The world production of pears in 2021 was 25.6 Mt across a cultivated area of 1.4 million hectares (ha) [3]. The largest part of pears are produced in Asia (72.2%) followed by Europe (15.1%), Americas (8.8%), Africa (3.2%), and Oceania (0.8%) [3]. In some producing areas, including Italy, pears growing is going through a moment of crisis due to biotic and abiotic stresses, the intensity of which is also increasing due to climate change. Indeed, emerging pests and diseases such as the Asian stink bug, blackspot, fire blight, psylla, pear scab, and *Valsa pyri* are increasing [4]. Moreover, over the last few years, winters have been too warm, with little or no rainfall, lack of snow, followed by late-frosts during the flowering and fruit-set period; the spring-summer periods have been characterized by long periods of very high maximum temperatures (>35 °C) and no rainfall, and therefore severe drought. The above-mentioned issues result in difficulties in the orchard management, reduced yield and efficiency, medium-small fruit sizes, and tree mortality. Regarding the latter, a sort of sudden pear decay was observed, mainly in high-density orchards of Abbé Fétel grafted on dwarfing quince clones [2].

In addition, there is a lack of varietal innovation in all the growing countries. Indeed, in Europe, the mostly diffused pear cultivar is Conference, followed by Abbé Fétel, Williams, and Rocha, while in Italy, one of the world’s largest producers of pears, a large part of production is based on two main cultivars, Abbé Fétel and Williams [2]. Among the cultivars that have been abandoned over the years due to changed cultivation requirements, there is the ‘Decana d’inverno’. This cultivar seems to be of Belgian origin, although it has been mainly used in France. Its spread was rapid throughout Europe and in a brief time the ‘Decana d’inverno’ also reached Italy, where its cultivation was recommended at the end of the 19th century [5]. This cultivar was particularly appreciated for its great fertility, rusticity, resistance to cold, and the quality of its fruit. Among the shortcomings reported by researchers there were the tendency of the fruit to crack when grown in a cold and humid exposure, the not always satisfactory vigour of the plant, and a marked tendency of the tree to age and decay, although there were century-old specimens still in production [5]. The spread of the ‘Decana d’inverno’ at the beginning of the 20th century was almost confined to the Piedmont region, although it was occasionally cultivated across northern Italy. Towards the end of the 20th century it was mainly cultivated in Emilia-Romagna, but with a limited overall importance [5]. In a context of lack of varietal innovation such as that of pear growing, the preservation of biodiversity is crucial, as varieties abandoned in the past could be carriers of interesting agronomic traits in facing the new scenario and the related challenges imposed by climate change. As proof of this, the ‘Decana d’inverno’ is now being used in breeding programmes. For example, Angelys^®^ was obtained in 1994 from the INRA by crossing ‘Decana del Comizio’ × ‘Decana d’inverno’, while ‘Cepuna’ was obtained by crossing ‘Conference’ and ‘Decana d’inverno’ [6]. In this context, an excellent tool for ex-situ conservation of tree species biodiversity is represented by *in vitro* culture [7,8]. *In vitro* cell and tissue culture techniques do indeed ensure the rapid multiplication and production of plant material under aseptic conditions and can be used for the conservation among the others of endangered and rare crops [9]. *In vitro* culture has been reported for several cultivars/rootstock of pear [10,11,12,13,14,15,16]. However, due to weak multiplication rate, hyperhydricity, and susceptibility to phenolic oxidation, pear is generally considered a recalcitrant species for *in vitro* propagation (micropropagation) [16,17]. In this context, there is a growing interest in the use of natural substances to improve the *in vitro* plant’s tissue culture [18]. Among the natural substances that can be used in the composition of *in vitro* growing media there is neem oil. Previous researches [19,20] demonstrated beneficial effects related to the use of neem oil for olive micropropagation, but no studies were conducted on *in vitro* culture of other plants species. Neem oil is obtained from *Azadirachta indica* A. Juss seeds and thanks to its composition its widely used in agriculture and for human health [21]. Neem oil contains at least one hundred of biologically active compounds represented mainly by triterpenes and among them azadirachtin is the main important [22]. In particular, in the agricultural sector it is used as pesticide, soil conditioner, and fumigant [21,23]. In this context, the aim of the present work was to evaluate the effect of adding neem oil to the growth substrate composition in order to optimise the *in vitro* culture of an ancient pear tree cultivar (‘Decana d’inverno’).

## 2. Results

**Figure 1:**
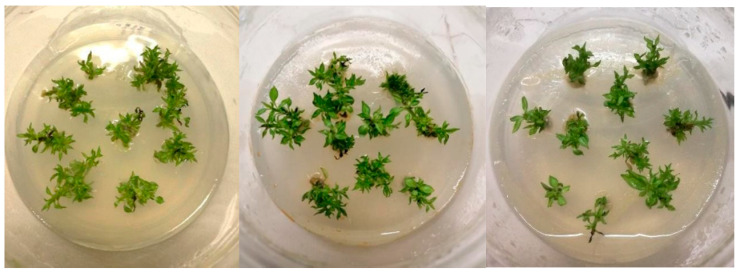
Overview of proliferated ‘Decana d’inverno’ shoots grown (from left to right) on substrate Control, ON 0.1 (with 0.1 mL L^−1^ neem oil added) and ON 0.5 (with 0.5 mL L^−1^ neem oil added).

**Figure 2:**
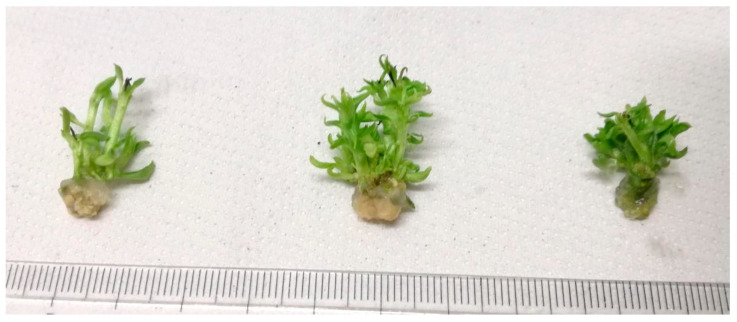
Proliferated explants grown (from left to right) on substrate Control, ON 0.1 (with 0.1 mL L^−1^ neem oil added) and ON 0.5 (with 0.5 mL L^−1^ neem oil added).

**Figure 3:**
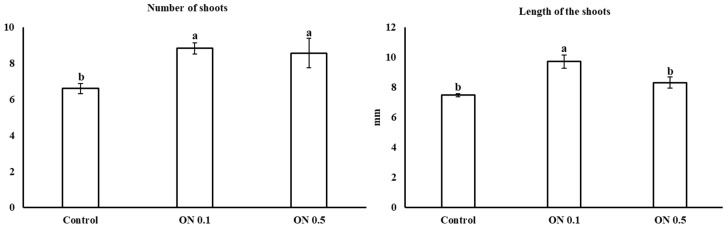
Means of number and length of shoots obtained in the three subsequent subcultures. Means values followed by different letters were significantly different (Tukey HSD test, *p* < 0.05).

Neem oil does not affect proliferated explant’s viability. In all treatments, the shoot viability of cv ‘Decana d’inverno’ was equal to 100%. Adding neem oil to the culture medium allowed to obtain a better vegetative activity evaluated as number of shoots produced (Figure 1 and Figure 2). The increase in the number of shoots produced by each initial explant was observed at both the concentration used (0.1 and 0.5 mL L^−1^) (Figure 3). Indeed, in the substrate where 0.1 mL L^−1^ (ON 0.1) and 0.5 mL L^−1^ (ON 0.5) of neem oil were added an increase of about 34% and 30% respect to the control (0 mL L^−1^ of neem oil) in the number of shoots was observed. On the contrary, a positive effect on length of shoots was detected only when neem oil was applied at 0.1 mL L^−1^. In the ON 0.1 substrate an increase in shoot length of about 30% was observed while at 0.5 mL L^−1^ neem oil did not induce an increase in shoot length.

The neem oil addition did not affect the percentage of explants with callus that was equal to 96.7%, 90% and 100% in control, ON 0.1 and ON 0.5, respectively. Moreover, the addition of neem oil to the culture media at 0.1 mL L^−1^ (ON 0.1) increased the callus fresh weight. On the contrary, the above-mentioned effect was not detected in the ON 0.5 culture media. The addition of neem oil did not affect green fresh weight and total dry weight of the proliferated explants (Table 1).

## 3. Discussion

Pear tree is generally considered recalcitrant for micropropagation since during *in vitro* culture it has some issues such as hyperhydricity, susceptibility to phenolic oxidation, and a low multiplication rate [16,17]. However, considering the advantages of micropropagation compared to other agamic propagation methods there is interest in developing protocols for pear *in vitro* culture, also as a tool for biodiversity conservation for ancient cultivars or particular genotypes with interesting traits. In the present work, an optimized multiplication protocol for pear was obtain through the use of neem oil. In particular, neem oil, especially at 0.1 mL L^−1^ improved shoots number and length, and this is very important because it allow to have easy-to-manage shoots in subculturing activity. A similar effect was observed *in vitro* on olive where neem oil improved shoots length, multiplication rate, and fresh and dry weights of the proliferated explants [19] and was able to replace zeatin partially [20]. To the best of our knowledge the mode of action of neem oil in plants was still not investigated. However, the neem oil effect is probably due to a combination of compounds and not a single compound [22]. Thanks to its complex chemical composition, neem oil can indeed act in synergy with the nutrient components of the growth medium, improving its trophic function and simulating the effects of gibberellins and cytokinins [19]. Improvement in pear micropropagation were obtained also using other techniques (i.e., light type) or growth regulators types and concentration in the substrates. In particular, improvements in the growth of cultivars William and San Giovanni were obtained using a LED rich in green and red wavelengths (AP67) [16] while in another study improvements for micropropagation of cv Arbi in terms of shoot length and leaf area were obtained using red light [11]. On the other hand regarding nutrient media composition, an optimization of *Pyrus communis* L., ‘Pyrodwarf^®^(S)’ rootstock *in vitro* proliferation was obtained thorough a proper combination of plant growth regulators in the MS substrate: a combination of 2 mg L^−1^ 6-Benzylaminopurine (BAP) and 1 mg L^−1^ Kinetin [10]. Evaluating different BAP and Thidiazuron (TDZ) concentration high proliferation rate (4.9) was obtained for pear cultivar ‘Bitterbirne Lukas’ on MS medium containing 4 mg L^−1^ BAP [13].

## 4. Materials and Methods

### 4.1. Plant Material, Neem Oil Treatments, and Growing Conditions

The plant material used in the experiment was derived from shoots of the proliferating cultivar Decana d’inverno present at the Laboratory of Micropropagation and *in vitro* Biotechnology of the Tree Science Research Unit of the Department of Agricultural Food and Environmental Sciences of the University of Perugia, where the experiment was conducted. The culture of ‘Decana d’inverno’ was stabilized aseptically, from meristematic apices taken from portions of vegetative resting branches, cut into pieces with a length of 3–4 cm (2–3 nodes), washed with running tap water, and immersed for 10 s in a solution of 70% ethyl alcohol with the addition of a few drops of surfactant (Tween 20) to remove surface impurities. The plant material was then immersed in a 2.5% sodium hypochlorite solution for 20 min and immediately transported under a laminar flow cabinet to operate under completely aseptic conditions. The explants were then washed twice in sterile distilled water to remove residues of the sterilizing agent. The meristematic apices of 0.3–0.5 mm length and approximately 0.1 mm in diameter were then removed under aseptic conditions using a stereomicroscope.

Finally, the apices were transferred into glass tubes each containing 5 mL of Murashige, and Skoog (MS) substrate [24], supplemented with sucrose (30 g L^−1^), hydrolyzed casein (100 mg L^−1^), agar provided by Duchefa (0.7 g L^−1^), pH of 5.5. Before exposing the apices to light, the tubes were covered with aluminium foil and kept in the dark in the growth chamber for 7 days to avoid photo-oxidation. The healthy (uncontaminated) apices were kept in the growth chamber at 22 ± 2 °C and gradually accustomed to light intensities of 40 µE m^−2^ s^−1^ and a 16-h photoperiod of light. The stabilization phase was completed after approximately 30–35 days. The obtained proliferated shoots were then transferred in glass vessels (500 mL capacity), each containing ten explants and 100 mL of MS substrate enriched with vitamin mixture of Jacquiot instead of MS vitamin mixture [25], gibberellic acid (GA3) (1 mg L^−1^), naphthalenacetic acid (NAA) (0.1 mg L^−1^) and BAP (2 mg L^−1^), sucrose (30 g L^−1^) and agar (7 g L^−1^), pH 5.5.

The initial explants for the experiment were represented by 10 mm about long uninodal explants deprived of the apical bud to maintain a high degree of uniformity. Glass vessels (500 mL capacity) were used in the experiment, each containing ten explants with two leaves and 100 mL of the above-mentioned substrate. Following previous experiences conducted on other species, three different neem oil concentrations (0 mL L^−1^ referred as ‘control’, 0.1 mL L^−1^ referred as ‘ON 0.1′, and 0.5 mL L^−1^ referred as ‘ON 0.5′) were added to the substrate before autoclaving at 115 °C for 20 min. In particular, a neem oil soluble in water provided by “Neem Italia company” composed by neem seed oil (from organic agriculture) and Polysorbate 85 was used. The placement of the explant inside the vessels occurred in aseptic conditions under a horizontal laminar flow cabinet. The explants were placed for 30 days in a growth chamber, characterized by a constant temperature of 22 ± 2 °C and a 16-h photoperiod of light with an intensity of 40 µE m^−2^ s^−1^.

### 4.2. Growth Parameters

For each treatment, a number of vessels (replicates) equal to six was foreseen: this allowed, at the end of the proliferation, to carry out the destructive measurements on the shoots of three pots and to assure the suitable number of explants for the next subculture.

To evaluate the effect of n.o. treatments, after 30 days, the following parameters for each of the three subsequent subcultures were monitored:

viability (%): incidence of green and viable explants;
shoots (n): average number of shoots developed by each initial explant (multiplication rate);
shoot length (mm): average length of developed shoots;
callus (%): incidence of explants that produced basal callus;
green fresh weight (mg): average fresh weight of developed vegetative organs (leaves, stems, buds);
callus fresh weight (mg): average fresh weight of callus masses that may have developed at the base of explants;
total dry weight (mg): average dry weight of the vegetative organs and callus, obtained by keeping the plant material in an oven for three days at 105 °C.

### 4.3. Statistical Analysis

The trial was organized according to a completely randomized design, with three treatments and 30 proliferated explants for each treatment and for each of the three subsequent subcultures. In particular, for each treatment and subculture, a number of vessels (replicates) equal to six was foreseen: at the end of the proliferation, this allowed to carry out the destructive measurements on the shoots of three pots (30 proliferated explants for each treatment) and to assure the suitable number of explants for the next subculture. Collected data were subjected to one-way variance analysis (ANOVA), and significant differences were assayed by Tukey HSD test (*p* = 0.05).

## 5. Conclusions

The obtained results suggest that the addition of neem oil, a natural substance, at 0.1 mL L^−1^, allows to optimize the *in vitro* culture of ‘Decana d’inverno’ an ancient pear tree cultivar. The increase in the number of shoots produced and their length allow to increase the efficiency of the micropropagation process and to overcome some issues of the *in vitro* culture of a species considered recalcitrant.

## Figures and Tables

**Table 1:** Means of green fresh weight, callus fresh weight, and total dry weight of the proliferated explants obtained in the three subsequent subcultures.

Treatment	Green Fresh Weight (mg)	Callus Fresh Weight (mg)	Total Dry Weight (mg)
Control	311.83 ± 12.86 a	80.69 ± 14.03 a	43.7 ± 5.32 a
ON 0.1	292.33 ± 39.79 a	113.58 ± 12.88 b	53.7 ± 4.21 a
ON 0.5	223.43 ± 19.93 a	62.03 ± 9.26 a	37.9 ± 4.32 a

Means values ± SE followed by different letters were significantly different (Tukey HSD test, *p* < 0.05).

## Data Availability

The data that support the findings of this study are available from the corresponding author upon reasonable request.
